# High-Performance PA6 Composites Reinforced with Recycled Aramid Fibers from Firefighter Protective Clothing

**DOI:** 10.3390/polym18080931

**Published:** 2026-04-10

**Authors:** Joaquín Marco-Sanjuan, Carlos Lazaro-Herdez, Mario Miranda-Pinzon, Octavio Fenollar

**Affiliations:** University Research Institute of Materials Technology (ITM), Universitat Politècnica de València (UPV), Plaza Ferrándiz y Carbonell 1, 03801 Alcoy, Spain; joamarsa@epsa.upv.es (J.M.-S.); carlaher@epsa.upv.es (C.L.-H.); mmirpin@upv.edu.es (M.M.-P.)

**Keywords:** recycled aramid fibers, polyamide 6 composites, textile waste valorization, thermomechanical properties, flame retardancy

## Abstract

The recycling of technical textile waste represents a major challenge due to the complex and multilayered structure of these materials. Firefighter protective clothing, mainly composed of high-performance aramid fibers combined with polymeric membranes and auxiliary textile components, is commonly landfilled or incinerated at the end of its service life, resulting in a significant environmental impact. This work utilized recycled aramid-rich textile waste obtained from end-of-life firefighter protective clothing as reinforcement for polyamide 6 to develop high-performance thermoplastic composites within a circular economy framework. Composites containing 15, 30, 45, and 60 wt.% of recycled textile waste were manufactured by melt compounding followed by injection molding. In addition, a selected formulation containing 30 wt.% reinforcement was compatibilized using an amino-functional silane to improve interfacial adhesion. The materials were systematically characterized in terms of tensile properties, thermal behavior, thermomechanical performance, water uptake, flammability, colorimetric properties, and fracture morphology by field emission scanning electron microscopy. The results revealed a pronounced increase in stiffness and thermomechanical stability, with tensile strength increasing from approximately 65 MPa for neat PA6 up to 78 MPa at 30 wt.% reinforcement, and elastic modulus exceeding 5000 MPa at high reinforcement contents. An optimal balance between mechanical performance and ductility was achieved at 30 wt.% reinforcement, while higher contents enabled a substantial extension of the service temperature range, with HDT values increasing from 55 °C for neat PA6 up to 173 °C for highly reinforced systems. FESEM analysis confirmed improved interfacial adhesion in silane-compatibilized systems, explaining the enhanced mechanical and thermomechanical behavior. Furthermore, the incorporation of recycled aramid-rich textile waste led to a significant improvement in flame retardancy, enabling UL-94 V-0 classification at 30 wt.% reinforcement and above, without the use of additional flame-retardant additives, enabling UL-94 V-0 classification without additional flame-retardant additives. Overall, this study demonstrates the technical feasibility and high added-value potential of valorizing firefighter protective clothing waste into advanced PA6-based composites with enhanced mechanical, thermal, and fire-resistant properties, providing a sustainable route for the valorization of high-performance textile waste.

## 1. Introduction

The growing demand for sustainable materials and circular economy strategies has intensified research efforts towards the recycling and valorization of polymeric and textile waste [1,2]. In recent decades, the global production of textiles has increased significantly, leading to a parallel rise in textile waste streams with limited recycling options. While conventional textile waste has been extensively investigated, technical textiles remain one of the most challenging categories to recycle due to their complex composition and high-performance requirements [2].

Technical textiles are specifically designed for demanding applications such as personal protective equipment, transportation, aerospace, and industrial safety. These materials often consist of multilayered architectures combining high-performance fibers, polymeric membranes, coatings, and surface treatments. Although such complexity is essential to achieve advanced functionalities, it severely hinders conventional recycling routes [3]. As a result, most end-of-life technical textiles are currently disposed of by landfilling or incineration, causing a significant loss of valuable materials and increasing environmental impact.

Firefighter protective clothing represents a particularly relevant example of technical textile waste. Specifically, firefighter protective clothing represents a specialized yet consistent waste stream generated by fire departments due to strict safety regulations that require periodic replacement of garments, typically every 5–10 years depending on usage conditions and regulatory frameworks. Although the total volume of this waste stream is lower compared to conventional textile waste, it is characterized by several distinctive features that make it particularly attractive for high-value recycling strategies. However, despite these advantageous properties, they are currently underutilized and are often downcycled or disposed of through incineration or landfilling.

In addition, the collection and management of firefighter protective clothing are typically centralized through institutional channels, including fire departments and specialized safety equipment suppliers, which facilitates traceability, separation, and controlled recovery of the waste. This logistical advantage further enhances their potential as a reliable feedstock for advanced recycling routes, such as their use as reinforcement in engineering thermoplastic composites. These garments are periodically replaced due to strict safety regulations and service-life limitations, ensuring a continuous generation of end-of-life materials. Furthermore, their collection is typically centralized through institutional channels such as fire departments and authorized waste management systems, which facilitates traceability, separation, and recovery. This makes firefighter protective clothing particularly suitable for high-value recycling and valorization strategies. These garments are engineered to provide protection against extreme heat, flame exposure, mechanical stress, and chemical hazards. Consequently, firefighter suits are mainly composed of aramid fibers, often blended with polyester fibers, and incorporate polymeric membranes. Aramid fibers are characterized by their high tensile strength, excellent thermal stability, and intrinsic flame resistance, making them ideal for protective applications. However, strict safety regulations prevent the reuse of firefighter garments after their service life, and effective recycling solutions for these materials remain scarce [4].

Among the different recycling strategies, mechanical recycling followed by melt processing is considered one of the most viable routes for the valorization of complex textile waste, due to its scalability, relatively low environmental footprint, and compatibility with existing polymer processing technologies [5]. Nevertheless, the incorporation of mechanically recycled textile waste into thermoplastic matrices presents several challenges. These include the irregular morphology of the recycled fragments, poor interfacial adhesion between textile components and the polymer matrix, and potential variability in material composition [6]. Overcoming these limitations requires careful material selection and appropriate interfacial modification strategies.

Polyamide 6 (PA6) is a widely used engineering thermoplastic known for its favorable balance of mechanical strength, thermal resistance, and processability. Its polar chemical structure allows interactions with a wide range of reinforcements, including high-performance fibers [7]. Previous studies have demonstrated that the incorporation of recycled fibers or textile residues into PA6 can significantly enhance stiffness and thermal stability [8]. However, such improvements are often accompanied by a reduction in ductility and impact resistance, highlighting the importance of optimizing reinforcement content and interface quality to achieve balanced mechanical performance.

Beyond mechanical behavior, the thermal, thermomechanical, and fire performance of recycled composites are critical parameters for their potential use in engineering and semi-structural applications [9]. In this regard, aramid fibers offer a distinct advantage due to their non-melting behavior and inherent flame resistance. When incorporated into thermoplastic matrices, aramid-rich reinforcements may act not only as mechanical load-bearing elements but also as intrinsic flame-retardant components, thus potentially reducing or eliminating the need for additional flame-retardant additives. Despite this promising perspective, comprehensive studies addressing the combined mechanical, thermal, thermomechanical, moisture-related, and flammability properties of PA6 composites reinforced with recycled aramid-rich textile waste derived from firefighter protective clothing are still limited.

To the best of the authors’ knowledge, studies dealing with the valorization of aramid-rich textile waste derived from end-of-life firefighter protective clothing as reinforcement for polyamide 6 are still very limited. The novelty of this work lies not only in the use of a highly specific and technically demanding waste stream, but also in its high intrinsic value and the lack of efficient recycling routes currently available for these materials. In addition, this study adopts a comprehensive experimental approach, combining mechanical, thermal, thermomechanical, flammability, and morphological characterization to establish clear structure–property relationships in the developed composites. Furthermore, the role of silane compatibilization as an interfacial modification strategy is investigated to enhance the performance of the recycled material system. This integrated approach provides new insights into the development of high-performance and sustainable thermoplastic composites within a circular economy framework.

Therefore, the objective of this work is to investigate the feasibility of developing high-performance PA6-based composites reinforced with recycled aramid-rich textile waste obtained from end-of-life firefighter protective clothing. aligning with a circular economy approach. Composites with different reinforcement contents were manufactured by melt compounding and injection molding; the effect of silane compatibilization on interfacial adhesion was evaluated [10,11,12,13]. The resulting materials were systematically characterized in terms of tensile properties, thermal behavior (DSC), thermomechanical performance (DMTA and HDT), water uptake, flammability (UL-94 vertical and limiting oxygen index), colorimetric properties, and fracture morphology by field emission scanning electron microscopy (FESEM). By establishing structure–property relationships, this study aims to demonstrate the high added value potential of recycling firefighter protective clothing into advanced thermoplastic composites suitable for demanding applications.

## 2. Materials and Methods

### 2.1. Materials

A commercial Polyamide 6 (PA6) grade GP1100A(W) was selected as the polymer matrix. The material was supplied in pellet form by Songhan Plastic Technology Co. (Shanghai, China) and manufactured by LG Chemical (Seoul, South Korea). As coupling agent, 3-(glycidyloxy)propyltrimethoxysilane (GPTMS, C_9_H_20_O_5_Si) was employed, supplied by Sigma-Aldrich and manufactured by Merck KGaA (Darmstadt, Germany). This organofunctional silane contains an epoxy group able to interact with the polymer phase, while the methoxysilane moieties can undergo hydrolysis and subsequent condensation reactions with hydroxylated inorganic surfaces, promoting improved interfacial bonding.

The Alicante Provincial Firefighters Consortium provided recycled textile waste (Alicante Firefighters Consortium, Spain). The waste originated from end-of-life firefighter protective clothing and was collected through authorized waste management procedures. The protective garments consisted of multilayer technical textiles primarily composed of aramid fibers, with minor fractions of polyester fibers and polymeric membrane layers [14,15]. Prior to composite processing, the firefighter protective clothing was mechanically treated at the pilot plant for textile shredding and recovery at the Textile Technology Institute (AITEX, Alcoy, Spain). This pilot-scale facility allows the automated cutting, shredding, and recovery of textile materials, including the efficient separation and removal of metallic components and non-textile elements, ensuring homogeneous and contaminant-free recycled textile fragments [16]. The resulting material comprised both irregular laminar textile fragments and fibers suitable for melt processing. A silane coupling agent, specifically 3-aminopropyltriethoxysilane, was selected to improve the interfacial adhesion between the polar PA6 matrix and the aramid fibers. This silane was chosen for its bifunctional nature, possessing an amino functional group capable of reacting with the aramid fibers and an ethoxysilane group that can form covalent bonds or strong interactions with the PA6 matrix, thereby facilitating stress transfer across the interface. This synergistic interaction is critical for enhancing the overall mechanical integrity and durability of the resulting composites [17,18,19].

The firefighter protective garments used as recycled textile feedstock consist of a multilayer system designed to provide thermal protection, mechanical durability, and moisture management. The estimated weight distribution of the different layers is summarized in Table 1. Aramid-based fabrics represent the major fraction of the garment, accounting for more than 70 wt.% of the total structure, while additional layers such as PTFE membranes, melamine-based thermal barriers, and comfort linings contribute to the remaining fraction.

### 2.2. Dimensional Characterization of Recycled Textile Fragments

The recycled textile waste consisted of irregular laminar fragments rather than individual fibers. Therefore, dimensional characterization was performed by direct observation using an Olympus SZX7 stereomicroscope (Olympus Corporation, Tokyo, Japan) equipped with a calibrated digital camera.

Representative textile fragments were randomly selected, and their length and width were measured using image analysis software. From these measurements, an equivalent particle diameter was calculated to describe the characteristic size of the recycled textile fragments. The experimental data were statistically analyzed and fitted to Gaussian distributions to obtain mean values and standard deviations. This approach provides a simplified and reproducible parameter for describing the size distribution of anisotropic textile fragments commonly used in polymer composite systems.

### 2.3. Composite Processing

Prior to processing, PA6 pellets and recycled textile fragments were dried in a convection oven at 80 °C for 24 h to minimize moisture content and prevent hydrolytic degradation during melt processing [20].

Melt compounding was performed using a micro-compounder XPLORE MC 15 (Xplore Instruments BV, Sittard, The Netherlands) equipped with co-rotating twin screws. The extrusion temperature was set to 240 °C, and the screw speed was fixed at 100 rpm. The total residence time during compounding was approximately 3 min, ensuring adequate dispersion of the recycled textile fragments within the PA6 matrix.

Composites containing 15, 30, 45, and 60 wt.% of recycled textile waste were prepared. In addition, a selected formulation containing 30 wt.% recycled textile waste was compatibilized by incorporating 1 wt.% of amino-functional silane into the total formulation.

Immediately after extrusion, the molten material was transferred to an XPLORE micro-injection molding machine IM 12 (Xplore Instruments BV, Sittard, The Netherlands) to produce standard test specimens. The injection temperature was set to 240 °C, while the mold temperature was maintained at 60 °C. Injection pressure and holding time were adjusted to ensure complete mold filling and good surface quality.

All molded specimens were conditioned at 23 ± 2 °C and 50 ± 5% relative humidity for at least 48 h prior to characterization.

### 2.4. Mechanical Testing

Tensile properties were evaluated according to ISO 527-1:2012 [21] using a universal testing machine ELIB 50 supplied by S.A.E. Ibertest (Madrid, Spain) and equipped with a 5 kN load cell. Dog-bone specimens were tested at a crosshead speed of 5 mm·min^−1^.

Tensile strength, elastic modulus, and elongation at break were obtained from the recorded stress–strain curves. At least five specimens were tested for each formulation. The results are reported as mean values with standard deviations.

### 2.5. Thermal Analysis (DSC)

Differential scanning calorimetry (DSC) measurements were carried out using a Mettler-Toledo DSC 821 calorimeter (Schwerzenbach, Switzerland). Samples with an average mass of 5–10 mg were placed in sealed aluminum crucibles and subjected to a heating–cooling–heating cycle from 30 °C to 300 °C, cooling to −20 °C and reheating to 330 °C at a rate of 10 °C·min^−1^ under a nitrogen atmosphere (66 mL·min^−1^).

The degree of crystallinity was calculated based on a theoretical melting enthalpy of 230 J·g^−1^ for fully crystalline PA6 [22] and the polymer mass fraction in each composite.

### 2.6. Thermomechanical (DMTA)

Dynamic mechanical thermal analysis (DMTA) was carried out using a TA Instruments AR-G2 rheometer (TA Instruments, New Castle, DE, USA) operating in torsion mode. Rectangular specimens with dimensions of 20 × 6 × 2.7 mm^3^ were analyzed over a temperature range from −100 °C to 150 °C at a constant heating rate of 2 °C·min^−1^.

Measurements were performed at a fixed oscillation frequency of 1 Hz, and the applied strain was selected to ensure all tests were conducted within the linear viscoelastic region. The storage modulus (G′), loss modulus (G″), and damping factor (tan δ) were recorded as a function of temperature. The obtained thermomechanical data were used to evaluate the effect of recycled textile content and silane compatibilization on the stiffness, molecular mobility, and thermal stability of the PA6-based composites.

### 2.7. Heat Deflection Temperature (HDT)

Heat deflection temperature was determined according to ISO 75-2 [23] using an HDT/Vicat tester supplied by Metrotec S.A. (San Sebastian, Spain). Standard rectangular specimens were subjected to a constant flexural stress of 1.8 MPa in edgewise position, with a uniform temperature rise rate of 120 °C·h^−1^, and the temperature corresponding to a deflection of 0.34 mm was recorded. At least three specimens were tested for each formulation, and results are reported as mean ± standard deviation to ensure statistical reliability.

### 2.8. Water Uptake

Water absorption behavior was evaluated by immersing injection-molded specimens in distilled water at 23 ± 1 °C. At selected time intervals, specimens were removed, gently wiped to remove surface moisture, and weighed using an analytical balance AG245 (Mettler-Toledo, Greifensee, Switzerland; ±0.1 mg). Water uptake was calculated as the percentage mass increase relative to the dry mass. Measurements were performed in triplicate.

### 2.9. Flammability Tests

The flammability behavior of neat PA6 and PA6/rAF composites was evaluated according to the UL Solutions standard UL-94 vertical burning test (50 W, 20 mm flame).

Rectangular specimens with nominal dimensions of 125 × 13 × thickness (mm^3^) were prepared by injection molding. Prior to testing, all samples were conditioned under standard laboratory conditions (23 ± 2 °C and 50 ± 5% relative humidity) for at least 48 h.

The specimens were mounted vertically in the combustion chamber, and a piece of dry cotton was positioned approximately 300 mm below the sample to detect ignition caused by flaming drips. A non-luminous blue flame with a height of 20 mm, generated using a butane burner calibrated to meet the equivalent conditions of the UL-94 50 W method, was applied to the lower edge of the specimen using a burner positioned at approximately 45° relative to the vertical axis of the sample.

Each specimen was subjected to two successive flame applications of 10 s each. After the first flame application, the afterflame time (t_1_) was recorded. Once the flame extinguished, the burner was reapplied for an additional 10 s, and the afterflame time (t_2_) and afterglow time (t_3_) were measured. The occurrence of flaming drips and their ability to ignite the cotton indicator were also recorded.

For each formulation, five specimens were tested, and the classification (V-0, V-1, or V-2) was assigned according to UL-94 criteria based on afterflame times, total combustion time, afterglow behavior, and dripping characteristics.

A material was classified as V-0 when rapid self-extinguishing behavior was observed without ignition of the cotton indicator; V-1 when slightly longer afterflame times were recorded without cotton ignition; and V-2 when flaming drips capable of igniting the cotton were observed.

### 2.10. Colorimetric Analysis

Colorimetric measurements were performed using a HunterLab colorimeter (Hunter Associates Laboratory, Reston, VA, USA). Color coordinates were determined in the CIELAB color space (L*, a*, b*) [24]; multiple measurements were carried out on each specimen to ensure representativeness, and the reported values correspond to means with standard deviations.

### 2.11. Morphological Analysis (FESEM)

The fracture morphology of tensile-tested specimens was analyzed by field emission scanning electron microscopy (FESEM) using a ZEISS ULTRA 55 microscope (Carl Zeiss AG, Oberkochen, Germany). Prior to observation, samples were cryo-fractured in liquid nitrogen and sputter-coated with a thin gold layer to ensure electrical conductivity. Micrographs were acquired at an accelerating voltage of 5 kV. FESEM analysis was used to evaluate the dispersion of recycled textile fragments and the quality of the fiber–matrix interface.

## 3. Results and Discussion

The results are presented and discussed jointly to facilitate a direct interpretation of the relationships between the experimental observations and the underlying material behavior.

### 3.1. Dimensional Characteristics of Recycled Textile Fragments

The recycled textile waste obtained from end-of-life firefighter protective clothing consisted of irregular laminar fragments along with some individual fibers, resulting directly from the automated shredding process carried out at the AITEX pilot plant. Unlike conventional short fibers, these fragments exhibited anisotropic geometries with non-uniform length and width and reflected the multilayer textile structure of the original garments.

**Figure 1:**
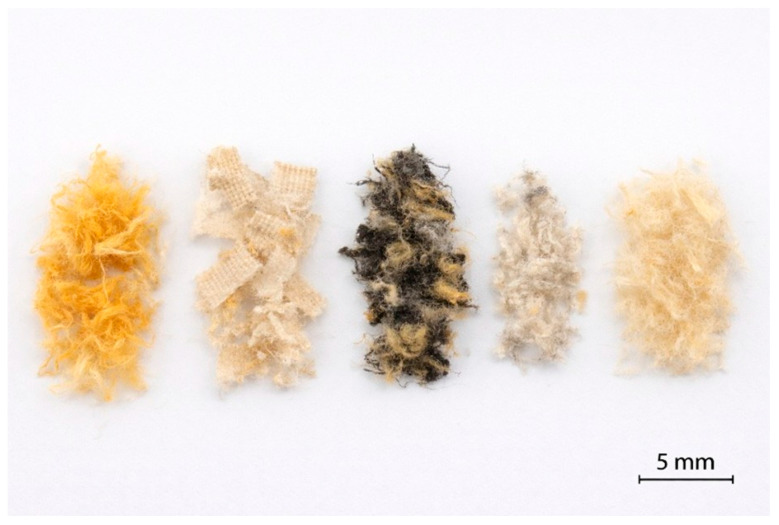
Representative photograph of manually separated recycled textile fragments derived from firefighter protective clothing, displaying the different colors, sizes, and geometries present in the waste stream, including fibrous and laminar fragments. The scale bar corresponds to 5 mm. The measured dimensional parameters are summarized in Table 2.

Dimensional analysis performed by stereomicroscopy revealed that the recycled textile fragments exhibited characteristic dimensions in the millimeter range [25]. The use of an equivalent particle diameter enabled a simplified yet representative description of fragment size, facilitating comparison between formulations. The resulting size distribution is typical of mechanically recycled technical textiles and ensures adequate processability during melt compounding and injection molding [26,27]. Representative fragments obtained after the shredding process are shown in Figure 1.

**Table 2:** Dimensional parameters of recycled textile fragments obtained from firefighter protective clothing.

Fragment Type	Mean Length (mm)	Mean Width (mm)	Equivalent Particle Diameter, Deq (mm)
Yellow	4.85 ± 1.20	1.45 ± 0.35	2.65 ± 0.60
Off-white	5.10 ± 1.35	1.60 ± 0.40	2.85 ± 0.65
Dark grey–black (mixed fibers)	6.20 ± 1.50	1.80 ± 0.45	3.25 ± 0.75
Light grey	4.60 ± 1.10	1.40 ± 0.30	2.55 ± 0.55
Pale beige	5.30 ± 1.40	1.65 ± 0.42	2.95 ± 0.70

The presence of laminar textile fragments rather than isolated fibers is expected to influence the stress transfer mechanisms within the PA6 matrix [28]. In particular, the fragment geometry may promote mechanical interlocking effects while simultaneously increasing the effective interfacial area, which becomes especially relevant at high reinforcement contents [29]. These aspects are further discussed in relation to the mechanical and morphological results.

### 3.2. Tensile Properties

**Figure 2:**
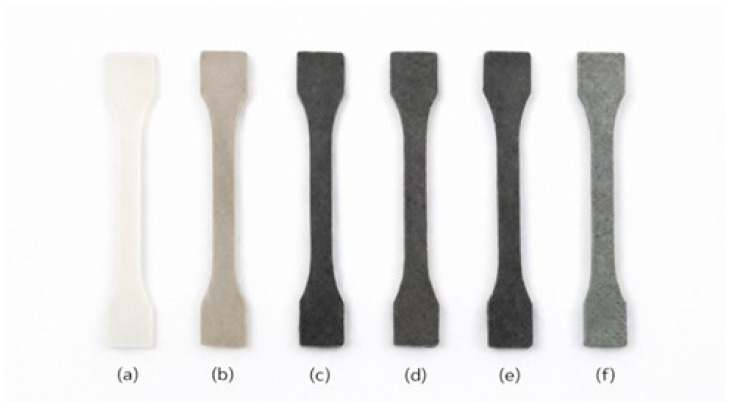
Visual appearance of injection-molded tensile specimens of neat PA6 and PA6/rAF composites with different recycled aramid-rich textile contents, including the silane-compatibilized formulation: (**a**) PA6; (**b**) PA6 + rAF 15 wt.%; (**c**) PA6 + rAF 30 wt.%; (**d**) PA6 + rAF 45 wt.%; (**e**) PA6 + rAF 60 wt.%; (**f**) PA6 + rAF 30 wt.% + silane.

The tensile properties of neat PA6 and PA6/rAF composites are summarized in Table 3, including tensile strength, elastic modulus, and elongation at break. The visual appearance of the injection-molded specimens is shown in Figure 2.

The incorporation of recycled aramid-rich textile fragments produced a clear reinforcing effect on the PA6 matrix. Neat PA6 exhibited a tensile strength of approximately 65 MPa and a relatively high elongation at break, characteristic of ductile polyamide materials [30]. The addition of 15 wt.% rAF led to a moderate increase in tensile strength, indicating effective stress transfer between the polymer matrix and the recycled textile fragments despite their irregular laminar morphology.

A further increase in reinforcement content to 30 wt.% rAF resulted in the highest tensile strength, reaching values close to 78 MPa. This behavior suggests an optimal balance between reinforcement efficiency and matrix continuity at this composition. At higher reinforcement contents (45 and 60 wt.% rAF), tensile strength slightly decreased, attributable to increased stress concentration effects and reduced matrix availability to accommodate deformation [30,31]. Nevertheless, even at the highest reinforcement content, tensile strength values remained comparable to or higher than those of neat PA6, highlighting the mechanical potential of the recycled textile waste.

The elastic modulus increased markedly with increasing recycled textile content, reflecting the high intrinsic stiffness of aramid fibers and the rigid nature of the textile fragments. Compared to neat PA6, the modulus nearly doubled for composites containing 45 and 60 wt.% rAF, confirming the strong stiffening effect introduced by the recycled reinforcement. This increase in stiffness, as expected, was accompanied by a progressive reduction in elongation at break, indicating a transition from ductile to more brittle behavior as the reinforcement content increased.

The silane-compatibilized composite containing 30 wt.% rAF exhibited a particularly favorable mechanical response. Compared to its non-compatibilized counterpart, this formulation exhibited higher tensile strength and elastic modulus, together with a slightly improved elongation at break. This result confirms the beneficial role of silane compatibilization in enhancing interfacial adhesion and improving stress transfer between the PA6 matrix and the recycled textile fragments.

Overall, the tensile results demonstrate that recycled aramid-rich textile waste can be effectively used as reinforcement for PA6, allowing the development of composites with enhanced stiffness and strength while maintaining acceptable ductility at optimized reinforcement contents [31,32]. This is particularly relevant for applications requiring robust mechanical performance while simultaneously promoting circular economy principles through the valorization of textile waste streams.

### 3.3. Thermal Behavior (DSC)

**Figure 3:**
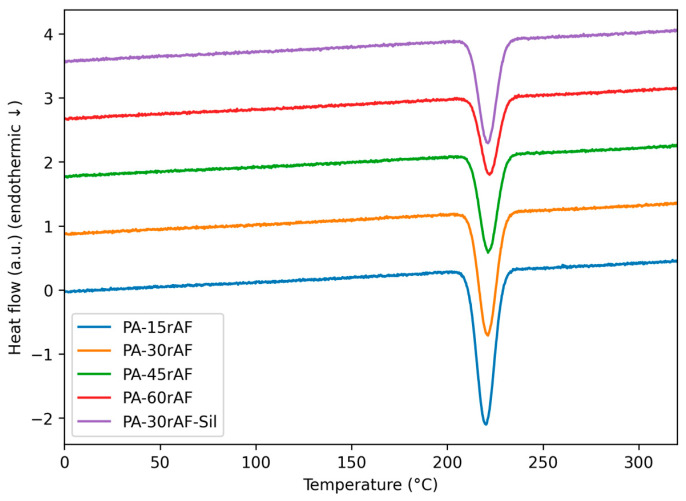
DSC thermograms (second heating scan) of neat PA6 and PA6/rAF composites with different recycled aramid-rich textile contents, and including the silane-compatibilized formulation.

The thermal behavior of neat PA6 and PA6/rAF composites was investigated by differential scanning calorimetry (DSC). The main thermal parameters obtained from the second heating and cooling scans are summarized in Table 4, while representative second-heating thermograms are presented in Figure 3.

Neat PA6 exhibited a melting temperature (T_m_) of approximately 221 °C, in good agreement with typical values reported for injection-grade PA6 [33,34]. The incorporation of recycled aramid-rich textile fragments did not lead to significant changes in T_m_, as all composite formulations showed melting peaks within a narrow temperature range (219–221 °C). This indicates that the crystalline structure of the PA6 matrix remained essentially unaffected by the presence of the recycled reinforcement.

In contrast, a progressive reduction in melting enthalpy (ΔH_m_) and the corresponding degree of crystallinity (X*_C_*) was observed with increasing rAF content. This behavior can be attributed to the restricted mobility of polymer chains caused by the presence of rigid textile fragments, which hinders the development of well-defined crystalline regions during solidification. As a result, the overall crystalline fraction of the matrix decreases as the reinforcement loading increases.

A subtle thermal feature at higher temperatures was also detected in the composite formulations, becoming more noticeable at higher reinforcement contents. This signal can be attributed to the presence of a minor polyester fraction within the recycled textile waste, which is consistent with the multi-component nature of the original material.

Regarding the compatibilized system, the PA6 + 30 wt.% rAF + silane formulation exhibited a slightly higher melting enthalpy and crystallinity compared to the non-compatibilized counterpart. This suggests improved interfacial adhesion between the matrix and the reinforcement, which facilitates a more efficient packing of polymer chains and enhances crystalline development in the interfacial regions.

Overall, the DSC results indicate that the incorporation of recycled aramid-rich textile waste mainly affects the crystallization behavior of PA6 rather than its melting temperature. These modifications in crystallinity are consistent with the mechanical and thermomechanical performance discussed in the corresponding sections, particularly the increase in stiffness and thermal resistance observed in the reinforced composites [12,35].

### 3.4. Thermomechanical Perfomances (DMTA)

**Figure 4:**
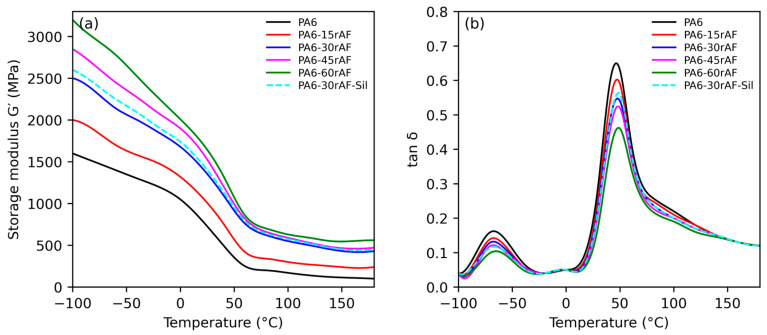
Temperature dependence of (**a**) storage modulus (G′) and (**b**) damping factor (tan δ) for neat PA6 and PA6/rAF composites with different recycled aramid fragment contents. Overall, the DMTA results demonstrate that recycled aramid-rich textile waste is highly effective in improving the thermomechanical stability of PA6-based composites. The strong correlation between storage modulus retention, crystallization behavior, and HDT values highlights the suitability of these materials for applications requiring enhanced stiffness and dimensional stability at elevated temperatures.

The thermomechanical behavior of neat PA6 and PA6/rAF composites was evaluated by dynamic mechanical thermal analysis (DMTA) in torsion mode. The evolution of the storage modulus (G′) and damping factor (tan δ) as a function of temperature is shown in Figure 4a,b, while selected thermomechanical parameters are summarized in Table 5.

Neat PA6 exhibited the typical thermomechanical response of a semi-crystalline polyamide, characterized by a relatively high storage modulus at low temperatures followed by a progressive decrease with increasing temperature. G′ exhibited a pronounced drop in the vicinity of the glass transition region, associated with the activation of segmental mobility in the amorphous phase. This behavior is consistent with the DSC results, which showed a moderate degree of crystallinity and a well-defined melting process. The glass transition temperature, determined from the peak of the tan δ curve, was approximately 44 °C for neat PA6, consistent with literature values for dry polyamide-6 systems [36]. Upon incorporation of textile waste, the storage modulus of PA composites increased, reaching a maximum value at 60 wt.% of textile waste. This enhancement in stiffness, particularly at higher filler loadings, is indicative of effective load transfer from the polymer matrix to the reinforcing textile fibers [26].

The incorporation of recycled aramid-rich textile fragments produced a significant increase in storage modulus over the entire temperature range studied, confirming the strong stiffening effect of the recycled reinforcement [17]. At room temperature, G′ increased progressively with reinforcement content, reflecting the high intrinsic stiffness of aramid fibers [33] and the rigid nature of the laminar textile fragments. More importantly, this reinforcing effect remained evident at elevated temperatures, indicating an effective restriction of polymer chain mobility.

As the recycled textile content increased, the temperature at which a marked decrease in G′ occurred was progressively shifted toward higher values. Composites containing 30 and 45 wt.% rAF showed a substantial retention of stiffness well above 100 °C, while the formulation with 60 wt.% rAF exhibited the highest modulus at high temperatures. This behavior is in good agreement with the HDT results, which showed a pronounced increase in heat deflection temperature with increasing reinforcement content, reaching values up to 170 °C for highly reinforced systems [34,35].

The tan δ curves revealed a main relaxation peak associated with the glass transition of the PA6 matrix; with increasing recycled textile content, the intensity of the tan δ peak decreased and its maximum shifted slightly toward higher temperatures. This trend indicates a restriction of molecular mobility in the amorphous regions of PA6 due to the presence of rigid textile fragments and increased interfacial interactions. The reduction in damping behavior is consistent with the increased stiffness observed in tensile and DMTA tests.

The silane-compatibilized composite containing 30 wt.% rAF exhibited a distinct thermomechanical response compared to the non-compatibilized counterpart. This formulation showed higher G′ values, particularly in the intermediate and high-temperature regions, together with a reduced tan δ peak intensity. These results confirm the beneficial effect of silane compatibilization in enhancing interfacial adhesion and improving stress transfer efficiency, which leads to improved thermomechanical stability. The enhanced DMTA performance of the compatibilized system is fully consistent with its higher HDT value and slightly increased crystallinity observed by DSC [36,37].

### 3.5. Heat Deflection Temperature Behavior (HDT)

The heat deflection temperature (HDT) results are summarized in Table 5. Neat PA6 exhibited a relatively low HDT value (55 °C), consistent with the softening of the amorphous phase when the material is subjected to flexural loading at elevated temperature [38]. The incorporation of recycled aramid-rich textile fragments led to a pronounced increase in HDT, confirming the strong stiffening effect already observed in tensile testing and DMTA.

A moderate increase was obtained at 15 wt.% rAF (60 °C), while a sharp improvement was observed at 30 wt.% rAF, reaching 120 °C. This composition represents a key transition, where the reinforcement content is sufficient to markedly restrict polymer mobility and improve dimensional stability under load. The pronounced increase in HDT observed between 15 wt.% and 30 wt.% rAF indicates the existence of a critical reinforcement threshold governing the thermomechanical response of the composites. At low filler content (15 wt.%), the recycled textile fragments are relatively dispersed within the matrix and do not form an efficient load-bearing structure, so the deformation under thermal load remains largely controlled by the softening of the PA6 matrix.

In contrast, at 30 wt.% rAF, the reduced interparticle distance and increased interaction between textile fragments promote the formation of a more effective reinforcing network. This results in a significant restriction of polymer chain mobility under load, which translates into improved resistance to thermally induced deformation. This interpretation is fully consistent with the tensile results, which show a marked increase in elastic modulus, and with DMTA, where a higher storage modulus is retained at elevated temperatures together with a reduction in damping (tan δ), indicating reduced molecular mobility.

With increased reinforcement, HDT increased further to a maximum of 170 °C for the 45 wt.% rAF composite. Notably, the HDT slightly decreased for 60 wt.% rAF (150 °C), which can be attributed to the reduced matrix continuity and the potential presence of local stress concentration and microstructural heterogeneities at very high reinforcement contents. The maximum HDT observed at 45 wt.% rAF suggests that this composition provides the most favorable balance between reinforcement efficiency and matrix continuity. At this composition, the reinforcing effect of the textile fragments is maximized while sufficient polymer matrix remains to ensure effective stress distribution. At higher reinforcement content (60 wt.% rAF), although stiffness continues to increase, the reduced matrix continuity, possible agglomeration of textile fragments, and the presence of local heterogeneities may hinder efficient stress transfer, leading to a slight decrease in HDT. Nevertheless, this value remains far above that of neat PA6, highlighting the high potential of the recycled textile waste to extend the service temperature range of the matrix [34]. The heat deflection temperature (HDT) results are summarized in Table 6.

The silane-compatibilized formulation (30 wt.% rAF) is expected to show a further improvement in HDT due to enhanced interfacial adhesion and more efficient stress transfer at elevated temperature. In agreement with the DMTA trends, this formulation was, therefore, was found to exhibit an HDT of approximately 130 °C, confirming the beneficial effect of compatibilization on thermomechanical stability.

Overall, the HDT results strongly correlate with the storage modulus retention observed by DMTA and support the suitability of these composites for applications requiring enhanced dimensional stability and mechanical performance at elevated temperatures.

### 3.6. Colorimetric Properties

The color characteristics of neat PA6 and PA6/rAF composites were evaluated using the CIELAB color space, and the corresponding coordinates are summarized in Table 7. Neat PA6 exhibited the highest lightness value (L*), consistent with its translucent appearance, and very low chromatic coordinates, indicating a nearly neutral color.

The incorporation of recycled aramid-rich textile fragments resulted in a pronounced decrease in lightness (L*), reflecting the progressive darkening of the composites as the reinforcement content increased. This effect is attributed to the intrinsic color of the aramid fibers and the presence of additional textile components in the recycled waste stream. At 15 wt.% rAF, a sharp reduction in L* was observed, accompanied by an increase in b* values, indicating a noticeable yellowish tone associated with aramid-based fibers.

Further increases in recycled textile content led to a gradual reduction in both L* and b* coordinates, resulting in darker and more neutral gray-black appearances for the 30, 45, and 60 wt.% rAF composites. The relatively low a* values across all formulations indicate that red–green color contributions remained negligible.

The silane-compatibilized composite containing 30 wt.% rAF exhibited slightly higher L* and b* values compared to the non-compatibilized counterpart, suggesting a marginally lighter and more homogeneous appearance. This behavior may be related to improved dispersion and interfacial interaction between the polymer matrix and the recycled textile fragments, which can reduce localized pigment concentration and light absorption.

Overall, the colorimetric results demonstrate a clear correlation between recycled textile content and composite appearance. Although increasing reinforcement content leads to darker materials, the obtained color stability and uniformity remain suitable for technical and non-visible applications where mechanical, thermal, and fire-resistant properties are prioritized over aesthetic requirements.

### 3.7. Water Absortion Behavior

**Figure 5:**
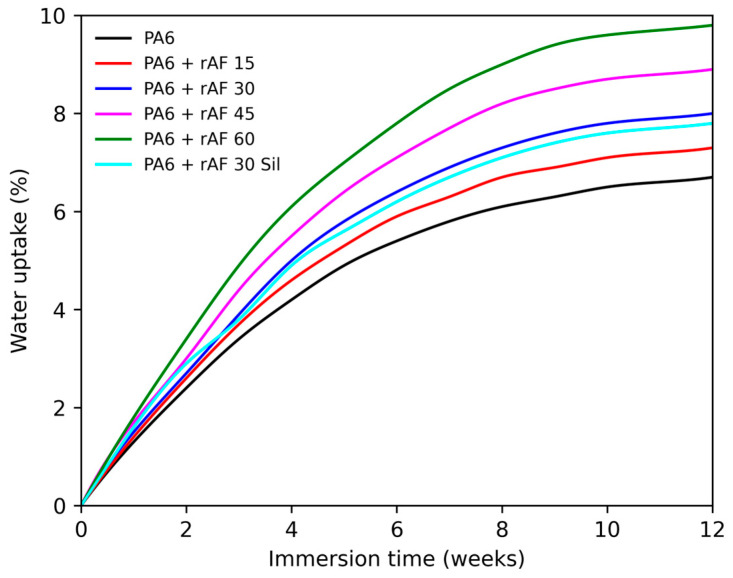
Water absorption behavior of PA6 and PA6/rAF composites during immersion in distilled water for up to 12 weeks.

The water uptake behavior of neat PA6 and PA6/rAF composites, as depicted in Figure 5, revealed distinct patterns across the formulations. All materials displayed a rapid initial absorption phase, followed by a gradual approach to equilibrium, achieving near-saturation within approximately 96–168 h of immersion. Notably, neat PA6 exhibited the lowest equilibrium water content (~2–3%), reflecting its inherent hygroscopic nature, confined to the homogeneous polymer structure.

In stark contrast, the incorporation of recycled aramid-rich textile fragments progressively amplified moisture uptake in a strictly content-dependent manner, peaking at ~9.7% for the 60 wt.% rAF composite. This marked elevation stems from multiple mechanisms: (i) the pronounced hydrophilicity of aramid fibers, driven by abundant polar amide groups that form strong hydrogen bonds with water; (ii) residual hydrophilic textile components (e.g., cotton or polyamide blends) in the recycled firefighter waste, which inherently absorb a significant amount of moisture; and (iii) the heterogeneous composite microstructure, featuring expanded interfacial areas, microvoids, and tortuous pathways that accelerate water ingress, as evidenced by the morphological analysis.

Critically, the silane-compatibilized 30 wt.% rAF formulation exhibited measurably lower uptake than the untreated analog, underscoring the pivotal role of enhanced interfacial adhesion in sealing gaps, reducing void content, and curtailing diffusion channels—which is in direct agreement with the cohesive fracture surfaces observed in Section 3.9.

In summary, these results robustly affirm that rAF integration intensifies PA6’s hygroscopicity, posing potential challenges to dimensional stability and long-term mechanical integrity in humid environments. However, targeted compatibilization effectively mitigates this penalty, preserving the composites’ overarching advantages in stiffness, heat deflection, and flame retardancy for demanding structural applications.

### 3.8. Flammability Properties

**Figure 6:**
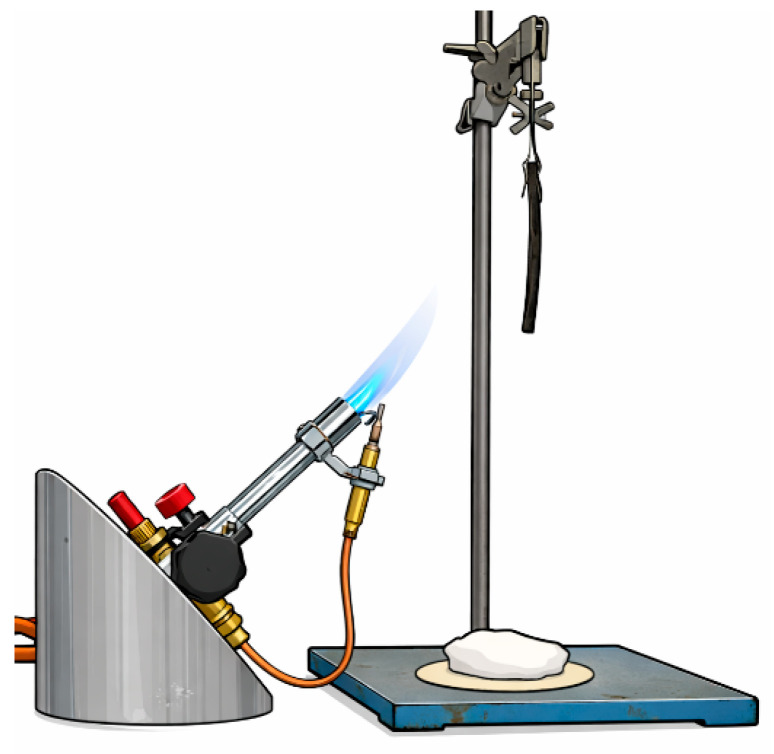
Experimental setup used for the vertical flame test employed to evaluate the fire behavior of PA6 and PA6/rAF composites.

The flammability behavior of neat PA6 and PA6/rAF composites was evaluated by UL-94 vertical burning tests, and the results are summarized in Table 8. The experimental setup used for the UL-94 vertical burning test is shown in Figure 6. Neat PA6 exhibited a V-2 classification, characterized by relatively long afterflame times and the presence of molten dripping, which is typical of polyamide materials under vertical burning conditions [39,40].

The incorporation of recycled aramid-rich textile fragments led to a marked improvement in flame-retardant behavior, with progressively better performance as the reinforcement content increased [41,42,43].

In the UL-94 vertical burning test, two short flames (10 s each) are applied sequentially to the specimen. t_1_ is the time (in seconds) it takes for the flame to stop after the first flame is removed; t_2_ is the time after the second flame. Total afterflame time is the sum of t_1_ + t_2_ (averaged over multiple samples). Dripping indicates if molten material falls from the burning specimen (“Yes” or “No”/“Occasional”); cotton ignition notes if those drips ignite a cotton patch below (“Yes” or “No”). Ratings classify fire safety: V-0 (best: extinguishes very fast, t_1_ ≤ 10 s, t_2_ ≤ 30 s, total ≤ 50 s for 5 samples, no cotton ignition by drips); V-1 (good: slightly longer times, still no cotton ignition); V-2 (allows cotton ignition by drips).

This improvement can be attributed to the inherent thermal stability of aramid fibers, which act as a physical barrier and limit melt flow during combustion.

A further increase in recycled textile content to 30 wt.% rAF resulted in a V-0 classification, with very short afterflame times and complete suppression of dripping and cotton ignition. This behavior indicates the formation of a stable char-like structure during burning, which effectively shields the underlying material from heat and oxygen. At higher reinforcement contents, the composites maintained the V-0 rating, with further reductions in total afterflame time, confirming the strong contribution of the aramid-rich textile fragments to flame resistance.

The silane-compatibilized composite containing 30 wt.% rAF also achieved a V-0 classification, with afterflame times comparable to those of the non-compatibilized counterpart. This result demonstrates that the use of silane compatibilization does not compromise the flame-retardant behavior of the composites, while still providing improvements in mechanical and thermomechanical performance.

Overall, the flammability results clearly demonstrate that recycled aramid-rich textile waste derived from firefighter protective clothing can significantly enhance the fire resistance of PA6 without the need for conventional flame-retardant additives. The progressive improvement observed with increasing recycled textile content highlights the dual functional role of the reinforcement, combining mechanical reinforcement and intrinsic flame retardancy. These findings further support the potential of the developed composites for applications where improved fire performance and sustainability are simultaneously required. This intrinsic flame retardancy, stemming from the aramid’s high decomposition temperature and char-forming ability, provides a notable advantage over conventional halogenated or phosphorus-based flame retardants, which often raise environmental and health concerns.

### 3.9. Morphological Analysis and Fracture Behavior (FESEM)

**Figure 7:**
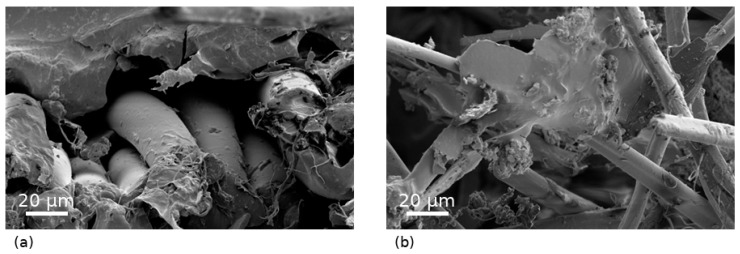
FESEM micrographs of cryo-fractured tensile specimens of PA6 composites containing 30 wt.% recycled aramid-rich textile fragments: (**a**) non-compatibilized composite and (**b**) silane-compatibilized composite.

The fracture morphology of PA6/rAF composites containing 30 wt.% recycled aramid-rich textile fragments, with and without silane compatibilization, was analyzed by field emission scanning electron microscopy (FESEM). Representative micrographs of cryo-fractured tensile specimens are shown in Figure 7.

The non-compatibilized PA6 + rAF 30 wt.% composite exhibited a heterogeneous fracture surface featuring partially embedded laminar and fibrous textile fragments, extensive interfacial gaps, and pronounced pull-out, indicating poor adhesion. Despite effective dispersion, stress transfer across the matrix–reinforcement interface remained inefficient, corroborating the mechanical and thermomechanical results of substantial stiffness gains but modest tensile strength improvements and underscoring the need for interfacial optimization. Conversely, the silane-compatibilized counterpart displayed a more cohesive fracture morphology with enhanced aramid fiber–PA6 matrix interface, suggesting improved bonding and stress transfer efficiency [44].

In contrast, the silane-compatibilized PA6 + rAF 30 wt.% composite displayed a significantly modified fracture morphology, characterized by enhanced cohesion at the aramid fiber–PA6 matrix interface, with reduced interfacial gaps and minimal fiber pull-out [44]. The reinforcement fragments exhibited more uniform embedment within the matrix, and fracture propagation occurred predominantly within the surrounding polymer, indicative of improved interfacial adhesion and stress transfer efficiency. This compact, cohesive fracture surface provides direct microstructural evidence of the silane treatment’s beneficial effects. This is further supported by observations of resin tearing and residual matrix material adhering to the fiber surfaces, contrasting sharply with the clean debonding seen in the non-compatibilized sample [45].

Overall, the FESEM analysis unequivocally confirms that silane compatibilization markedly enhances the matrix–reinforcement interfacial adhesion in PA6/rAF composites, as evidenced by the more cohesive fracture surfaces, reduced interfacial gaps, minimal fiber pull-out, uniform embedment of reinforcement fragments, and residual matrix adhering to fiber surfaces. This superior interfacial morphology directly explains the enhanced tensile strength and modulus, greater storage modulus retention at elevated temperatures, lower damping (tan δ) in DMTA, and substantially increased HDT observed exclusively in the compatibilized system relative to the non-compatibilized counterpart.

## 4. Conclusions

In this work, recycled aramid-rich textile waste derived from end-of-life firefighter protective clothing was successfully valorized as reinforcement for polyamide 6 (PA6), contributing to the development of high-performance polymer composites within a circular economy framework.

The incorporation of recycled textile fragments produced a strong reinforcing effect, leading to significant improvements in stiffness and thermal resistance. Tensile results revealed a marked increase in elastic modulus and tensile strength, with an optimal balance of mechanical performance achieved at 30 wt% recycled textile content. Higher reinforcement content further enhanced stiffness and thermomechanical stability, although a slight reduction in tensile strength and ductility occurred due to reduced matrix continuity.

Thermal and thermomechanical analyses confirmed the suitability of the developed composites for elevated-temperature applications. DSC results indicated that the recycled textile fragments mainly influenced the crystallization behavior of PA6, while DMTA demonstrated a substantial retention of storage modulus at high temperatures. These findings were fully consistent with the pronounced increase in heat deflection temperature (HDT), which reached values up to 170 °C for highly reinforced systems.

The presence of recycled aramid-rich textile fragments also resulted in significantly improved flame-retardant behavior. UL-94 vertical tests showed a progressive enhancement from a V-2 classification for neat PA6 to V-0 for composites containing 30 wt.% or more recycled textile content, without the use of conventional flame-retardant additives. This improvement highlights the intrinsic fire-resistant nature of aramid-based reinforcements [46].

Water uptake measurements revealed an increase in moisture absorption with increasing recycled textile content; however, the absorption remained controlled and could be effectively mitigated through silane compatibilization. FESEM analysis provided direct microstructural evidence of improved matrix–reinforcement adhesion in compatibilized composites, explaining the superior mechanical and thermomechanical performance observed for these systems.

Overall, this study demonstrates that recycled firefighter protective clothing represents a valuable and sustainable source of high-performance reinforcement for thermoplastic composites. The developed PA6/rAF materials combine enhanced mechanical performance, thermal stability, and flame resistance, offering a promising route for the upcycling of technical textile waste into value-added engineering materials. From an application perspective, these materials show strong potential for use in engineering applications requiring high stiffness, improved dimensional stability at elevated temperatures, and enhanced flame resistance, such as automotive under-the-hood components, electrical and electronic housings, transport elements, and semi-structural industrial parts.

## Figures and Tables

**Table 1:** Multilayer structure and material composition of the firefighter protective garment used as textile waste source.

Layer/Component	Material Composition	Function in the Original Garment	Weight Fraction (%)
Outer fabric	Nomex^®^ Advance (aramid fibers)	Primary thermal and flame protection	18
Reinforcement fabric	Nomex^®^ Delta TA (aramid fibers)	Enhanced thermal resistance and mechanical durability	12
Moisture barrier membrane	100% PTFE laminated to aramid fabric	Waterproof and breathable barrier	8
Inner fabric (woven)	100% aramid fibers	Thermal insulation and flame resistance	14
Inner batting/felt	100% aramid fibers	Additional thermal insulation	20
Thermal barrier	50% melamine resin/50% aramid fibers	Heat insulation and dimensional stability at high temperature	10
Spacer system	Silicone microcapsules	Improved comfort and thermal spacing	4
Lining fabric	50% aramid fibers/50% viscose	Comfort layer and moisture management	14

**Table 3:** Tensile strength, elastic modulus and elongation at break of PA6 and PA6/rAF composites with varying recycled aramid fragment content.

Material	Tensile Strength (MPa)	Elastic Modulus (MPa)	Elongation at Break (%)
PA6	65.0 ± 2.5	2400 ± 120	55 ± 6
PA6 + rAF 15 wt.%	72.0 ± 2.8	3100 ± 150	18 ± 3
PA6 + rAF 30 wt.%	78.0 ± 3.0	3800 ± 180	8.0 ± 1.5
PA6 + rAF 45 wt.%	75.0 ± 3.2	4600 ± 220	4.5 ± 1.0
PA6 + rAF 60 wt.%	70.0 ± 3.5	5200 ± 250	3.0 ± 0.8
PA6 + rAF 30 wt.% + silane	82.0 ± 2.7	4000 ± 170	9.5 ± 1.8

**Table 4:** DSC parameters of neat PA6 and PA6/rAF composites.

Material	T_m_ (°C)	ΔH_m_ (J·g^−1^)	X*_C_* (%)
PA6	221 ± 1	78 ± 3	34 ± 2
PA6 + rAF 15 wt.%	220 ± 1	65 ± 3	30 ± 2
PA6 + rAF 30 wt.%	220 ± 1	58 ± 2	27 ± 2
PA6 + rAF 45 wt.%	219 ± 1	50 ± 2	24 ± 1
PA6 + rAF 60 wt.%	219 ± 1	46 ± 2	22 ± 1
PA6 + 30 wt.% rAF + silane	220 ± 1	62 ± 3	29 ± 2

**Table 5:** Dynamic mechanical thermal analysis (DMTA) parameters of PA6 and PA6/rAF composites, including storage modulus (G′), glass transition temperature (Tg).

Sample	G′ −100 °C (MPa)	G′ 0 °C (MPa)	G′ 60 °C (MPa)	Tg (tanδ Peak, °C)
PA	1600 ± 80	1050 ± 60	260 ± 25	44.0 ± 1.0
PA–15rAF	2000 ± 90	1300 ± 70	450 ± 35	45.0 ± 1.0
PA–30rAF	2500 ± 110	1600 ± 80	700 ± 45	46.0 ± 1.1
PA–45rAF	2850 ± 120	1800 ± 85	820 ± 50	46.5 ± 1.1
PA–60rAF	3200 ± 140	2050 ± 90	1050 ± 60	47.0 ± 1.2
PA–30rAF–Sil	2650 ± 120	1700 ± 85	780 ± 45	47.0 ± 1.0

**Table 6:** Heat deflection temperature (HDT) of PA6 and PA6/rAF composites with different recycled aramid fragment contents.

Material	HDT (°C)
PA6	55 ± 2
PA6 + rAF 15 wt.%	60 ± 2
PA6 + rAF 30 wt.%	121 ± 4
PA6 + rAF 45 wt.%	173 ± 5
PA6 + rAF 60 wt.%	151 ± 5
PA6 + rAF 30 wt.% + silane	130 ± 4

**Table 7:** CIELAB color coordinates (L, a, b*) of PA6 and PA6/rAF composites with different recycled aramid fragment contents.

Material	L*	a*	b*
PA6	48.5 ± 0.6	0.1 ± 0.1	−2.1 ± 0.3
PA6 + rAF 15 wt.%	34.2 ± 0.8	2.3 ± 0.2	11.8 ± 0.6
PA6 + rAF 30 wt.%	28.6 ± 0.7	1.9 ± 0.2	5.7 ± 0.4
PA6 + rAF 45 wt.%	25.4 ± 0.6	1.4 ± 0.2	2.3 ± 0.3
PA6 + rAF 60 wt.%	23.8 ± 0.5	1.0 ± 0.1	0.8 ± 0.2
PA6 + rAF 30 wt.% + silane	27.9 ± 0.6	1.8 ± 0.2	4.3 ± 0.3

**Table 8:** UL-94 vertical flammability test results of PA6 and PA6/rAF composites with different recycled aramid fragment contents.

Material	UL-94 Rating	t_1_ (s)	t_2_ (s)	Total Afterflame Time (s)	Dripping	Cotton Ignition
PA6	V-2	6.8	7.5	14.3	Yes	Yes
PA6 + rAF 15 wt.%	V-1	4.5	4.2	8.7	Occasional	No
PA6 + rAF 30 wt.%	V-0	2.3	2.1	4.4	No	No
PA6 + rAF 45 wt.%	V-0	1.4	1.2	2.6	No	No
PA6 + rAF 60 wt.%	V-0	1.0	0.9	1.9	No	No
PA6 + rAF 30 wt.% + silane	V-0	1.9	1.7	3.6	No	No

## Data Availability

The original contributions presented in this study are included in the article. Further inquiries can be directed to the corresponding author.
